# Decoding Reveals Plasticity in V3A as a Result of Motion Perceptual Learning

**DOI:** 10.1371/journal.pone.0044003

**Published:** 2012-08-28

**Authors:** Kazuhisa Shibata, Li-Hung Chang, Dongho Kim, José E. Náñez, Yukiyasu Kamitani, Takeo Watanabe, Yuka Sasaki

**Affiliations:** 1 Department of Cognitive, Linguistic & Psychological Sciences, Brown University, Providence, Rhode Island, United States of America; 2 Division of Social and Behavioral Science, Arizona State University, Phoenix, Arizona, United States of America; 3 Brain Information Communication Research Laboratory Group, Advanced Telecommunications Research Institutes International, Kyoto, Japan; Nothwestern University, United States of America

## Abstract

Visual perceptual learning (VPL) is defined as visual performance improvement after visual experiences. VPL is often highly specific for a visual feature presented during training. Such specificity is observed in behavioral tuning function changes with the highest improvement centered on the trained feature and was originally thought to be evidence for changes in the early visual system associated with VPL. However, results of neurophysiological studies have been highly controversial concerning whether the plasticity underlying VPL occurs within the visual cortex. The controversy may be partially due to the lack of observation of neural tuning function changes in multiple visual areas in association with VPL. Here using human subjects we systematically compared behavioral tuning function changes after global motion detection training with decoded tuning function changes for 8 visual areas using pattern classification analysis on functional magnetic resonance imaging (fMRI) signals. We found that the behavioral tuning function changes were extremely highly correlated to decoded tuning function changes only in V3A, which is known to be highly responsive to global motion with human subjects. We conclude that VPL of a global motion detection task involves plasticity in a specific visual cortical area.

## Introduction

Adults can show significant improvements after training on various visual tasks, and such training effects are called visual perceptual learning (VPL) [1]. It has been found VPL is often specific for a visual feature trained or presented in training. Such specificity is observed in behavioral tuning function changes with the highest improvement centered on the trained visual feature and was originally thought to be evidence for changes in early visual system associated with VPL [2].

However, neural loci of VPL are highly controversial [1], [3]. It has been reported that VPL involves plasticity in lower visual areas such as V1 [4], [5], [6], [7], [8] or higher visual areas such as V4 [9], [10], [11] and MT/MST [12], [13]. On the other hand, recent single-unit recording and neuroimaging studies suggest that VPL does not involve changes in sensory tuning function of visual areas [14], but rather reflects changes in the process to read out sensory representation by decision-related cortical area such as lateral intraperietal area (LIP) [15] and anterior cingulate cortex (ACC) [16].

The controversy concerning whether VPL involves sensory plasticity in visual areas seems to be mainly due to lack of strong efforts to extensive comparison between changes in a behavioral tuning function and changes in a neural tuning function in multiple visual areas after VPL training. Single-unit recording has shown changes in neural tuning functions of a trained visual feature only in one or two areas [6], [15]. Functional magnetic resonance imaging (fMRI) can measure neural activation changes in multiple visual areas. Previous fMRI studies in VPL usually assumed that changes in amplitudes of fMRI signals after VPL training is correlated with changes in a behavioral tuning function. However, our recent fMRI study showed that this strong assumption is not always correct [17]. In addition, using conventional amplitude-based methods, S/N ratio of fMRI signals are not sufficiently high to produce reliable tuning functions [18]. To get around these restrictions, we used a decoding method [18], [19] to compute more reliable tuning functions from patterns of fMRI signals without the performance-amplitude assumption. We used the decoding method to compare changes in behavioral tuning function and decoded tuning functions in as many as 8 visual areas in association with VPL. Moreover, we carefully controlled subjects’ attention throughout the fMRI experiments so that the attention effects should not confound the fMRI activation.

After VPL training using a global motion detection task, we found that decoded tuning function changes only in V3A, which is known to be highly responsive to global motion processing for humans [20], [21], [22], were significantly and also highly correlated with behavioral tuning function changes. Our finding indicates that VPL in the global motion detection task is associated with sensory plasticity at least in a specific area of human visual cortex.

## Results

Six subjects participated in this study. The entire experiment consisted of a 12-day behavioral session preceded and followed by 1-day fMRI sessions (pre-fMRI and post-fMRI stages). In a separate experiment, retinotopy was measured to identify individual cortical visual representations for each subject (see Figure S1 for retinotopic map of a representative subject) using a standard protocol [23], [24], [25], [26], [27], [28].

**Figure 1:**
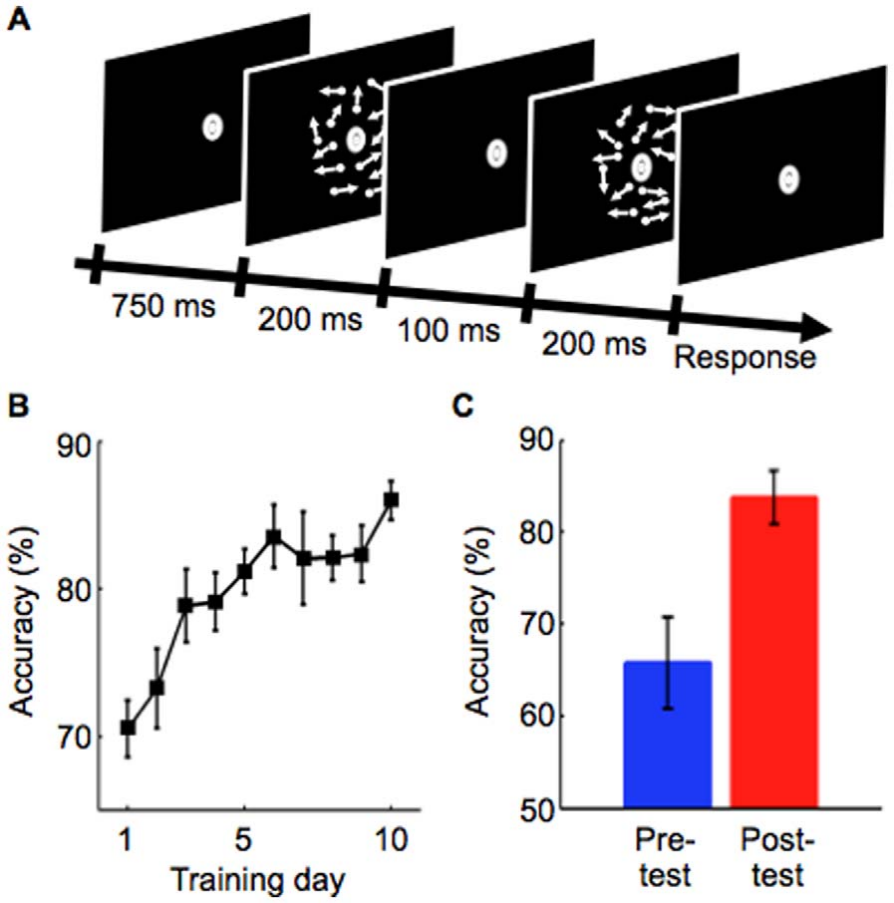
Task procedure and results of the behavioral session. (A) The 2IFC global motion detection task in the behavioral session. One stimulus interval contains 15% coherent motion while the other interval contains random motion (0% coherence). After two motion presentations, the subjects were asked to report the interval (first or second) which contained 15% coherent motion. (B) Mean performance across the subjects during the training stage. A motion direction used in this stage was defined as a trained direction for each subject. After 10-day training, a significant training effect was obtained (day 1 vs day 10, paired t-test, *P*<10^−4^). Error bars represent SEM. (C) Mean behavioral performance across the subjects for the trained direction in the pre-test (blue) and post-test (red) stages. Significant performance improvement was found after the training stage (paired t-test, *P = *0.01). Error bars represent SEM.

As a training task, we used a two interval-forced-choice (2IFC) global motion detection task, which is known to generate direction specificity of learning [29]. After two motion presentations in each trial, the subjects were asked to report the interval (first or second) which contained 15% coherent motion (Figure 1A).

The behavioral session consisted of a 10-day training stage preceded and followed by 1-day test stages (pre- and post-test stages). In the test stages, subjects’ performance on the 2IFC motion detection task for 9 directions (−48, −36, −24, −12, 0, 12, 24, 36, 48 deg from a designated motion direction for each subject) were measured to obtain a behavioral tuning function. In the training stage, the subjects conducted the 2IFC motion detection task in each day only using the designated motion direction as a trained direction. After the 10-day training stage, subjects’ performance for the trained direction significantly improved by 15% on average (Figure 1B; day 1 vs. day 10, paired t-test, *P*<10^−4^). Thus, we compared subjects’ performance for the trained direction between the pre- and post-test stages. Consistent with the performance improvement observed in the training stage, a significant improvement for the trained direction was found (paired t-test, *P = *0.01; Figure 1C), indicating that VPL for the trained direction occurred as a result of training on the motion detection task.

In the pre- and post-fMRI stages, we measured subjects’ brain responses to random motion (0% coherence) and the 9 motion directions used in the test stages of the behavioral session with 50% coherence while the motion stimuli were task-irrelevant. During fMRI measurement, a fixation task was used to control effects of subjects’ attention. Every time a white central fixation point turned to faint pink in an unpredictable timing manner, the subjects were asked to immediately press the button on a box in their right hand. To test whether subjects’ attention depends on the presented motion type (10 types in total; the random motion and the 9 motion directions) and the behavioral training session, we classified each color change of the fixation point according to the motion type presented when the color change occurred and calculated performance on the color change task in each day of the fMRI sessions (pre- and post-fMRI stages). No significant effect of motion type, fMRI stage, or interaction of these two factors on subjects’ attention was found in hit rate (two-way ANOVA with repeated measures, *P*>0.35; Figure S2A) and the number of false alarm (*P*>0.46; Figure S2B). These results indicate that subjects’ attention level was kept constant throughout the fMRI measurements and was not biased by any motion direction including the trained direction.

**Figure 2:**
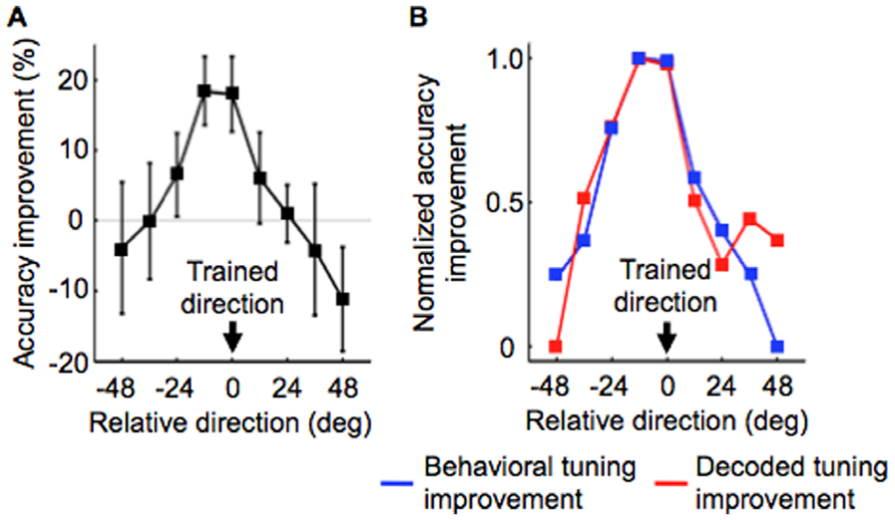
Comparing the two tuning improvement functions. (A) A behavioral tuning improvement function. The behavioral tuning improvement function was defined by mean performance change across the subjects between the pre- and post-test stages for each of 9 motion directions. Error bars represent SEM. (B) Comparison between the behavioral tuning improvement function (blue) and decoded tuning improvement function for V3A (red). Each improvement function was scaled from 0 to 1 for visualization purpose. Between the two improvement functions, a significant correlation was found (*r = *0.86, *P*<0.05, multiple correction by the number of ROIs with false discovery rate).

To explore visual area(s) that exhibit changes correlated with VPL found in the behavioral session, we specified 8 regions of interests (ROIs) in the visual cortex (V1, V2, V3, VP, V3A, V4v, V4d, and MT+) according to an individual retinotopic map for each subject (see Figure S1 for retinotopic map of a representative subject). We performed a decoding analysis [18], [19] on a pattern of fMRI signals for each ROI. For each of 9 motion directions, a linear decoder was trained to distinguish coherent motion from random motion. A decoding accuracy was calculated by evaluating performance of the decoder in a cross-validation framework.

First, we tested which ROI reflects the VPL that was observed in the behavioral session (Figure 1C). If a ROI reflects the behavioral VPL, then the decoding accuracy for the trained direction should be increased after the training in that ROI. We compared the decoding accuracies for the trained direction in the pre- fMRI and post-fMRI stages in each ROI. Results of t-tests for the ROIs revealed that only V3A showed a significant increase in the decoding accuracy for the trained direction (paired t-test, *P*<0.05, false discovery rate, corrected by the number of the ROIs). No significant improvement for the trained direction in the decoding accuracy was found for any of the other ROIs (*P*>0.1, no multiple correction).

**Figure 3:**
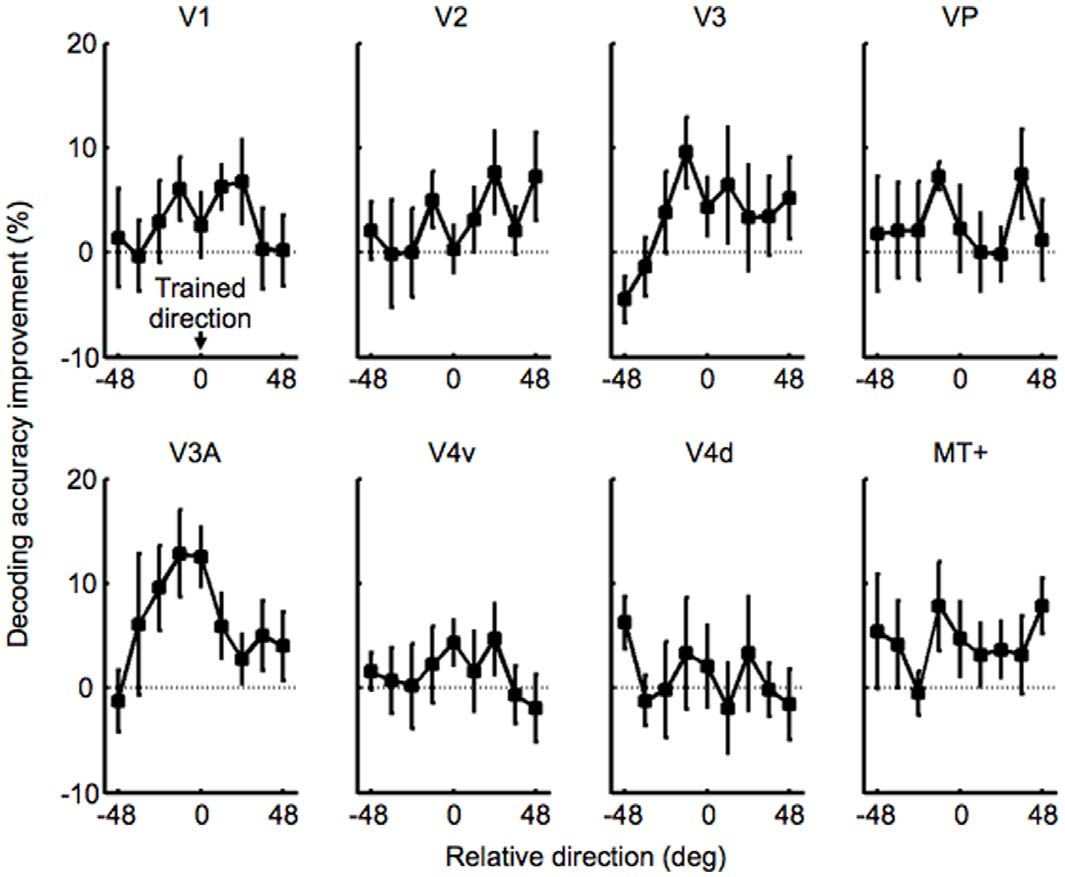
A decoded tuning improvement function for each ROI. Mean decoding performance improvement across the subjects was calculated by subtracting decoding accuracy in the pre-fMRI stage from that in the post-fMRI stage for each of 9 motion directions. Significant improvement for the trained direction was obtained only in V3A (paired t-test, *P*<0.05, false discovery rate, corrected by the number of the ROIs). Error bars represent SEM.

Next, we examined which ROI shows a high correlation between changes in the behavioral tuning functions and the decoded tuning functions after the behavioral training. To do so, we calculated a correlation coefficient between a behavioral tuning improvement function and a decoded tuning improvement function in each ROI. The behavioral tuning improvement function was defined as subtraction of the behavioral tuning function in the pre-test stage from that in the post-test stage (Figure 2A; see Figure S3 for the behavioral tuning functions for the pre- and post-test stages). We defined subtraction of a decoded tuning function in the pre-fMRI stage from that in the post-fMRI stage as the decoded tuning improvement function (Figure 3), where the decoded tuning function was calculated by decoding accuracies for the 9 motion directions (see Figure S4 for the decoded tuning functions for the pre- and post-fMRI stages). Then, we calculated the correlation coefficient between the behavioral tuning improvement function and the decoded tuning improvement function for each ROI. Only V3A showed a significant correlation (Figure 2B; *r = *0.86, *P*<0.05, false discovery rate, corrected by the number of ROIs). This tendency was also found when we transformed the two tuning improvement functions into t-values and calculated the correlation coefficient, considering inter-subject variability (*r = *0.84, *P*<0.05, false discovery rate, corrected by the number of ROIs). On the other hand, we found no significant correlation for the other ROIs (*P*>0.11, no multiple correction; see Figure S5 for comparison between the behavioral and decoded tuning improvement functions for each ROI and Figure S6 for its correlation coefficient). Thus, only V3A reflected VPL for the trained direction observed in the behavioral session and showed the decoded tuning improvement function correlated with the behavioral tuning improvement function.

## Discussion

In the present study, we systematically explored over the visual cortex to determine the visual area in which activation can explain behavioral performance enhancement in association with VPL of a global motion detection task by comparing the decoded tuning improvement functions of 8 visual areas with the behavioral tuning improvement function. Our recent study have shown that changes in amplitudes of fMRI signals do not necessarily correlate with the degree of behavioral improvement during VPL training [17]. Thus, in the present study we used the decoding method on patterns of fMRI signals to calculate the decoded tuning improvement functions rather than using amplitudes of fMRI signals. We found that only area V3A showed a significant decoded tuning improvement function and also that the decoded tuning improvement function of V3A was highly correlated with the behavioral tuning improvement function. As indicated in the Introduction, it is highly controversial whether sensory plasticity occurs within the visual cortex in association with VPL [4], [5], [6], [7], [8], [9], [10], [11], [12], [13], [15], [16]. Our results support the idea that VPL is associated with changes in the visual cortex, since the motion direction tuning function of V3A changed in association with VPL.

In our results, the decoded tuning improvement function in MT+ was not significantly correlated with the behavioral tuning improvement function. Such a lack of signature of VPL in MT+ is consistent with the study that measured neurons only in MT and LIP of monkeys [15]. Our previous fMRI study showed that human V3A differentially responds to different types of global motion but MT+ does not show such selectivity [22]. V3A involvement in VPL of the global motion detection task is probably due to this selectivity to global motion.

We found that only V3A showed a significant change in the decoded tuning function and a remarkable correlation with the behavioral VPL. However, it should be noted that absence of significant changes in the decoding tuning in the other ROIs than V3A does not necessarily indicate no involvement in these ROIs in VPL. In fact, V1 and V3 showed a limited but certain degree of improvements in the decoding accuracy for the trained direction (Figure 3 and S4) and of mild correlation with the behavioral tuning improvement function (Figures S5 and S6) although they were not statistically significant. Our results do not exclude the possibility of involvement in these ROIs in association with motion VPL while V3A showed the most prominent change in our decoding approach. In any case, our findings indicate that plasticity in the visual cortex can occur in association with VPL.

One might argue that the improvement in the decoding accuracy for the trained motion direction (Figure 3, V3A) is simply due to change in attention for the direction. However, it is highly unlikely because of the following aspects. First, it has been reported that when attention activates a visual area, it does not only the area itself but also higher visual areas [30]. However, decoding accuracy changes for the trained direction were obtained only in V3A. Second, we observed no significant change in performance on the central fixation task across the presented motion directions in the pre- and post-fMRI stages (Figure S2). This result suggests that subjects’ attention was equally engaged in the fixation task across different motion directions. Based on these points, we conclude that it is unlikely that the improvement in the decoding accuracy is attributed merely to changes in attention to the trained direction.

In summary, using a decoding technique, we examined changes in direction tuning functions in 8 visual areas in association with VPL to get around the problems that conventional fMRI analyses cannot provide clear neural tuning functions and that single-unit studies provides neural tuning functions at most in a few areas. The results indicate that VPL of a global motion detection task is associated with sensory plasticity in the visual cortex such as V3A. Future studies will be required to systematically address whether different stimuli or training tasks in VPL involves different neural mechanisms or cortical areas.

## Materials and Methods

The entire experiment consisted of a 12-day behavioral session preceded and followed by 1-day fMRI sessions (pre-fMRI and post-fMRI stages). The mean interval (±SEM) between the pre- and post-fMRI stages were 12±2 days.

### Subjects

Six naïve subjects (21 to 32 years; 2 males and 4 females) participated in the study. The study was approved by Institutional Review Boards of the Massachusetts General Hospital and Boston University. All subjects gave written informed consents and had normal or corrected-to-normal vision.

### Apparatus

Visual stimuli were presented on a LCD display (Viewsonic, VA2226w, 1680×1050 resolution, 60 Hz refresh rate) in the behavioral session and via LCD projector (Sharp, Note Vision6, 1024×768 resolution, 60 Hz refresh rate) during fMRI measurements in a dim room using Psychtoolbox 3 (http://psychtoolbox.org) on Mac OSX.

### Visual Stimuli and Task

Random dot motion was presented as a visual stimulus within an annulus subtending from 1.5 to 10 deg diameter on a black background. Dot density was 0.91 dots per deg^2^. The motion display consisted of coherent motion and random motion. Dots that composed the coherent motion are called signal dots and those that moved randomly are called noise dots. In each frame of 16.7 ms, each white dot (0.3 deg square) was randomly classified into either signal or noise. Signal dots moved to a predetermined direction at the speed of 24 deg per second, and noise dots were allocated in random positions. For example, for the 15% coherence level, 15% of the dots in the motion display moved to the predetermined direction from one frame to the next and then a different set of dots moved to that direction in the next frame transition [31].

We used a two interval-forced-choice (2IFC) global motion detection task, which is known to generate direction specificity of learning [29]. In each trial, the subjects performed the 2IFC motion detection task, in which one interval contains random motion (0% coherence) while the other interval contains 15% coherent motion (Figure 1A). The interval during which the coherent motion was contained was counterbalanced across trials. Throughout the task, the subjects were asked to fixate on a white bull’s eye fixation point on a gray disc (1.5 deg diameter) at the center of the display. Each trial started from a 750-ms fixation period. After presentation of two 200-ms motion displays separated by a 100-ms blank period, the subjects were asked to report which interval contained the coherent motion, by pressing one of two buttons on a keyboard. If the button press did not occur within two seconds, the trial was terminated. For each subject, only several trials were discarded due to the termination on each day. After each trial, a 500-ms inter-trial interval was inserted.

### Behavioral Session

The behavioral session consisted of a 10-day training stage preceded and followed by 1-day pre- and post-test stages. In the test stages of the behavioral session, subjects’ performance on the 2IFC global motion detection task for 9 directions (−48, −36, −24, −12, 0, 12, 24, 36, 48 deg from a designated motion direction) were measured. The order of the motion directions was counterbalanced across trials. The designated motion directions were off-cardinal directions (68, 113, 158, 248, 293, 338 deg) and counterbalanced among the subjects. Before the onset of the pre-test stage, the subjects were afforded a brief practice session. During the test stages, each subject completed 30 trials of the task for each of the 9 directions (about 20 minutes). After every 45 trials, the subjects were provided a brief rest period. During the training stage, the subjects performed the same task as in the test stages. Only the designated direction was presented as a trained direction for each subject. No accuracy feedback was given to the subjects. Each subject completed 720 trials in each day of the training stage (about 45 minutes). The subjects were provided a brief rest period after every 45 trials.

### FMRI Session

The pre- and post-fMRI stages consisted of a main experiment and a visual field localizer experiment. In the main experiment, subjects’ blood-oxygen-level-dependent (BOLD) signals were measured for random motion (0% coherence) and the 9 motion directions presented in the behavioral session with 50% coherence using the same motion algorithm. Each run consists of 20 stimulus periods preceded and followed by 6-second blank periods. In each stimulus period, one of 10 motion types (random dot motion and 9 motion directions) was presented for 6 seconds in pseudo-random order. Each subject participated in 24 runs. After each run, a brief rest period was provided according to the subjects’ request.

Throughout the run, the subjects were asked to report changes in color of the central fixation point by pressing the button in their right hand as soon as they detected the changes. In an unpredictable timing manner, the fixation color turned from white to faint pink during a 750-ms time window. Each subject’s response within 750 ms was regarded as a hit, and a response outside of this time window was regarded as a false alarm. The mean (±SEM) number of the fixation color changes was 41.8±1.1 in each run.

In the same session of the main experiment, the subjects were presented a reference stimulus to localize the retinotopic regions corresponding to the stimulated visual field in the main experiment. The ‘visual field localizer’ was composed of random motion (0% coherent motion) presented within an annulus subtending from 2 to 9.5 deg diameter for 12 seconds, interleaved with 12-second fixation periods. Subjects participated in two runs of 240 seconds. We used a smaller annular region for the visual field localizer than for the stimulus used in the main experiment to avoid selecting voxels corresponding to the stimulus edge, which may contain information irrelevant to motion direction [19]. During the visual field localizer experiment, the subjects performed the same fixation task as in the main experiment.

In other experiments, standard retinotopic mapping [23], [24], [25], [26], [27] and MT+ localization [28] procedures were completed to delineate visual areas on flattened cortical representations.

The subjects were scanned in a 3T MR scanner (Siemens, Trio) with a head coil. Functional MR images were acquired using gradient EPI sequences for measurement of BOLD signals. For the main and visual field localizer experiment, 33 contiguous slices (TR = 2 seconds, TE = 30 ms, flip angle = 90 deg, voxel size = 3×3×3.5 mm^3^) oriented parallel to the AC-PC plane were acquired, covering the entire brain. For the retinotopy measurement, 25 contiguous slices (TR = 2 seconds, TE = 30 ms, flip angle = 90 deg, voxel size = 3×3×3 mm^3^) oriented vertical to the Calcarine sulcus were acquired to cover the occipital cortex. T1-weighted MR images (MP-Rage; TR = 2.531 second, TE = 3.28 ms, flip angle = 7 deg, 256 slices, voxel size = 1.3×1.3×1 mm^3^, resliced during analysis to 1 mm^3^) were acquired for use in subsequent reconstruction of cortex in flattened format [32], [33].

### Regions of Interests (ROIs)

To explore visual area(s) that exhibit decoded tuning function changes correlated with behavioral tuning function changes, we specified 8 regions of interests in the visual cortex: V1, V2, V3, VP, V3A, V4v, V4d, MT+. These ROIs were defined using an individual retinotopic map [23], [24], [25], [26], [27] for each subject (see Figure S1 for retinotopic map of a representative subject). For all ROIs, left and right hemispheres were merged.

### FMRI Data Analysis

Data processing was conducted using FS-FAST and FreeSurfer software (http://surfer.nmr.mgh.harvard.edu/). All functional images were motion corrected [34] and registered to the individual anatomically reconstructed brain.

For data in the visual field localizer experiment, signal intensity of functional images was normalized individually across runs. No spatial smoothing was applied. Estimated BOLD signal amplitude and its t-value were computed based on a univariate general linear model for each voxel.

The data samples used for decoding analysis were created by the following steps. First, the voxels were sorted according to their amplitudes (t-values) to the visual field localizer within each ROI. For each ROI, we selected the most significant 100 voxels for the decoding analysis, as decoding accuracy had saturated at this pattern size across ROIs, resulting in a dimensionality compatible with previous studies [18], [35], [36]. Second, a time-course of BOLD signal intensity in the main experiment was extracted in each selected voxel and underwent linear trend removal for each run. Third, the time-course of BOLD signal intensity for each voxel was shifted by 2 volumes (4 seconds) to account for the hemodynamic delay, and then averaged in each 6-second stimulus period. Finally, the time-course of each voxel was normalized (z-score) separately for each run to minimize baseline differences across runs. The normalized and time-averaged BOLD signal intensity of each voxel was used as the data sample for decoding analysis.

We used a linear support vector machine (SVM) [37] in SVM-KM toolbox (http://asi.insa-rouen.fr/enseignants/~arakotom/toolbox/index.html) as a decoder and a leave-one-run-out cross-validation procedure to evaluate the decoder’s performance [18]. For each motion direction, we trained the decoder to associate a pattern of BOLD signals with a label (random motion or coherent motion) using 92 data samples (46 samples for random motion, 46 samples for coherent motion) from 23 runs. We then calculated performance (percent correct) of the decoder by testing whether the decoder predicted the stimulus (random motion or coherent motion) using independent data samples (two samples for random motion, two samples for coherent motion) from a remaining run.

## Supporting Information

Figure S1 **A retinotopic map on the flattened right hemisphere of a representative subject.** Yellow and blue colors indicate representations of the horizontal and vertical medians, respectively.
(TIFF)

Figure S2 **Performance of the fixation task in the fMRI sessions.** (A) Mean hit rate across the subjects for each of 10 motion types (the random motion and the 9 motion directions) in the pre- (blue) and post- (red) fMRI stages. RND stands for a random motion. No significant effect of motion type, fMRI stage, and interaction of these factors was observed (two-way repeated measures ANOVA, *P*>0.35). Error bars represent SEM. (B) The mean number of false alarm across the subjects for each of 10 motion types (the random motion and the 9 motion directions) in the pre- (blue) and post- (red) fMRI stages. No significant effect of motion type, fMRI stage, and interaction of these factors was observed (*P*>0.46). Error bars represent SEM.
(TIFF)

Figure S3 **Mean behavioral tuning functions across the subjects in the pre- (blue) and post- (red) test stages.** Error bars represent SEM.
(TIFF)

Figure S4 **Mean decoded tuning functions across the subjects in the pre- (blue) and post- (red) fMRI stages for each ROI.** Error bars represent SEM.
(TIFF)

Figure S5 **Comparison between the behavioral (blue) and decoded (red) tuning improvement functions for each ROI.** Each improvement function was scaled from 0 to 1 for visualization purpose. Only for V3A, a significant correlation was found (*r = *0.86, *P*<0.05, false discovery rate, corrected by the number of the ROIs), but not for the other ROIs (*P*>0.11, no multiple correction).
(TIFF)

Figure S6 **The correlation coefficient between the behavioral and decoded tuning improvement functions for each ROI.**
(TIFF)
