# Supplementary figures and images for: Decoding Reveals Plasticity in V3A as a Result of Motion Perceptual Learning
Source: PLoS One. 2012 Aug 28;7(8):e44003. doi: 10.1371/journal.pone.0044003 (PMC3429406; doi:10.1371/journal.pone.0044003)

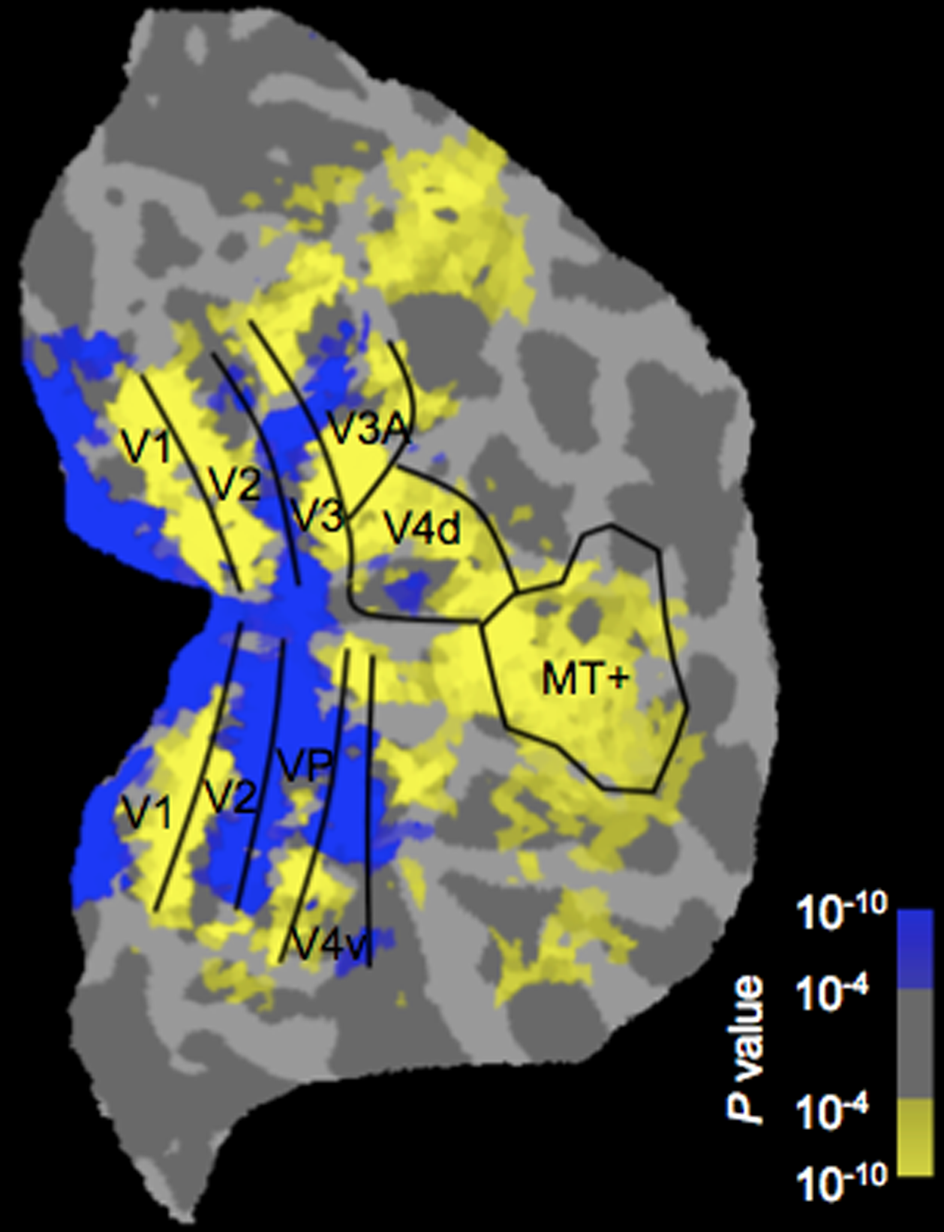

Supplement: Figure S1 — A retinotopic map on the flattened right hemisphere of a representative subject. Yellow and blue colors indicate representations of the horizontal and vertical medians, respectively. (TIFF) [file pone.0044003.s001.tif]

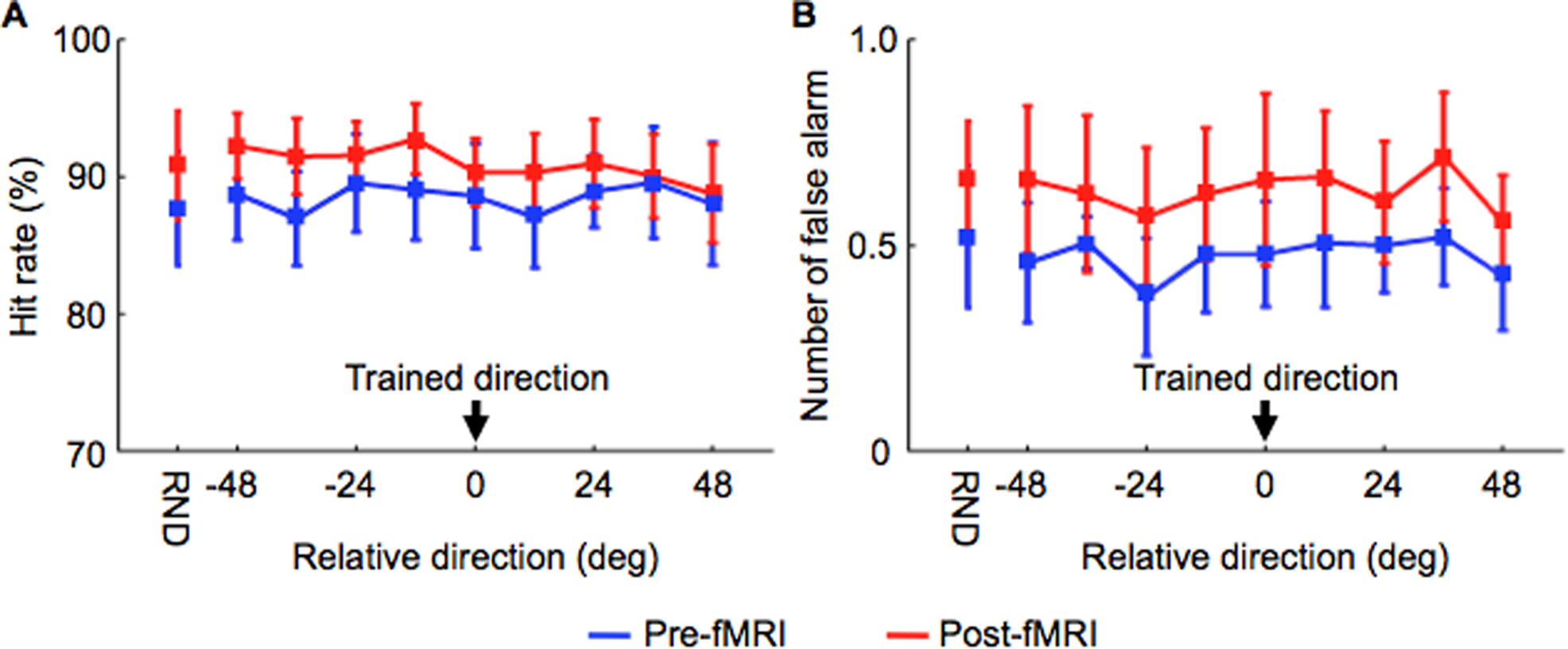

Supplement: Figure S2 — Performance of the fixation task in the fMRI sessions. (A) Mean hit rate across the subjects for each of 10 motion types (the random motion and the 9 motion directions) in the pre- (blue) and post- (red) fMRI stages. RND stands for a random motion. No significant effect of motion type, fMRI stage, and interaction of these factors was observed (two-way repeated measures ANOVA, P>0.35). Error bars represent SEM. (B) The mean number of false alarm across the subjects for each of 10 motion types (the random motion and the 9 motion directions) in the pre- (blue) and post- (red) fMRI stages. No significant effect of motion type, fMRI stage, and interaction of these factors was observed (P>0.46). Error bars represent SEM. (TIFF) [file pone.0044003.s002.tif]

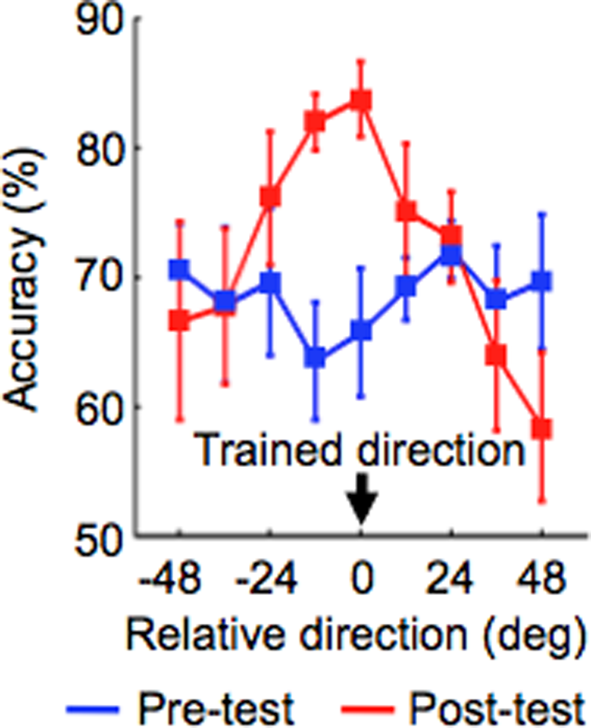

Supplement: Figure S3 — Mean behavioral tuning functions across the subjects in the pre- (blue) and post- (red) test stages. Error bars represent SEM. (TIFF) [file pone.0044003.s003.tif]

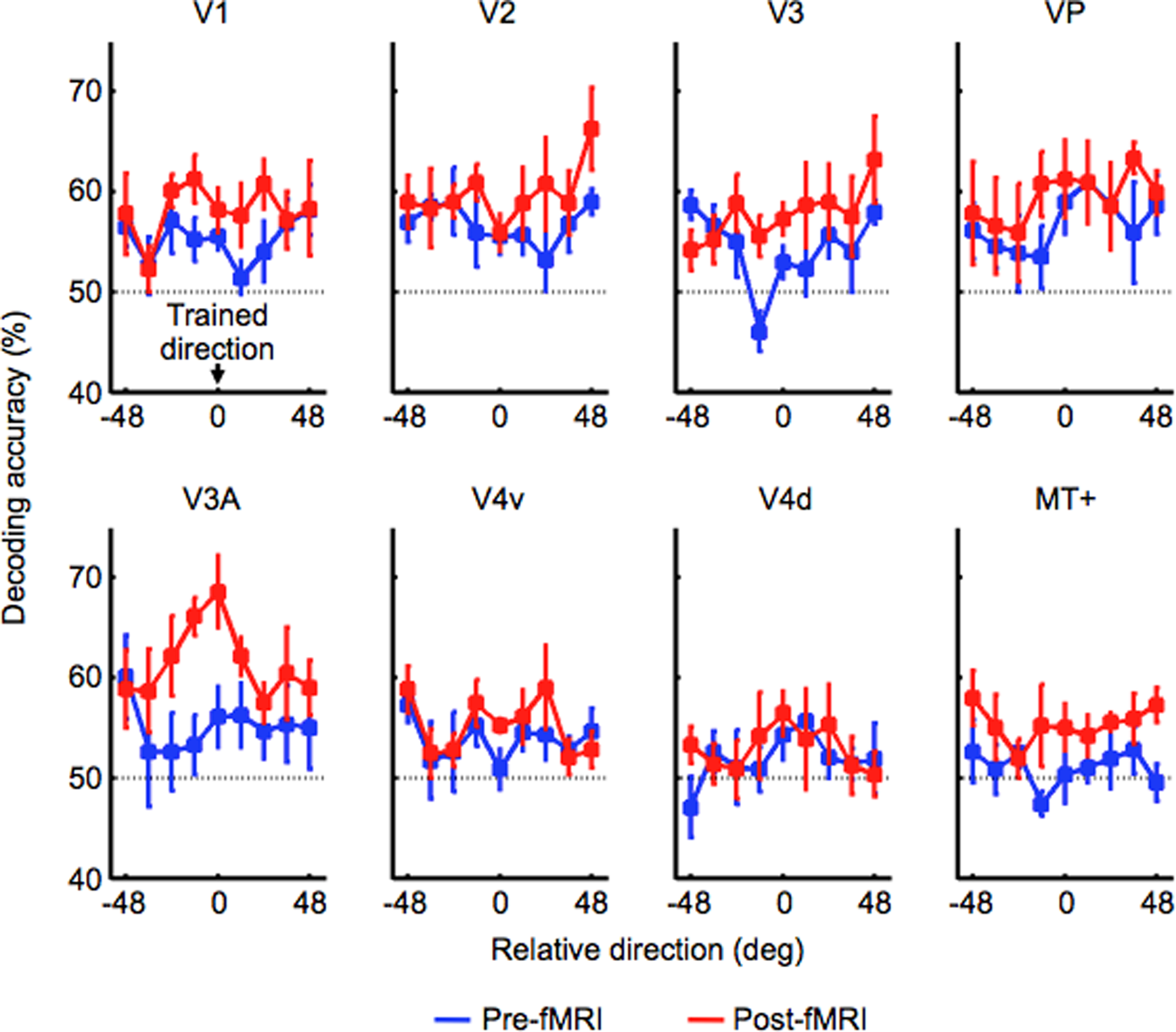

Supplement: Figure S4 — Mean decoded tuning functions across the subjects in the pre- (blue) and post- (red) fMRI stages for each ROI. Error bars represent SEM. (TIFF) [file pone.0044003.s004.tif]

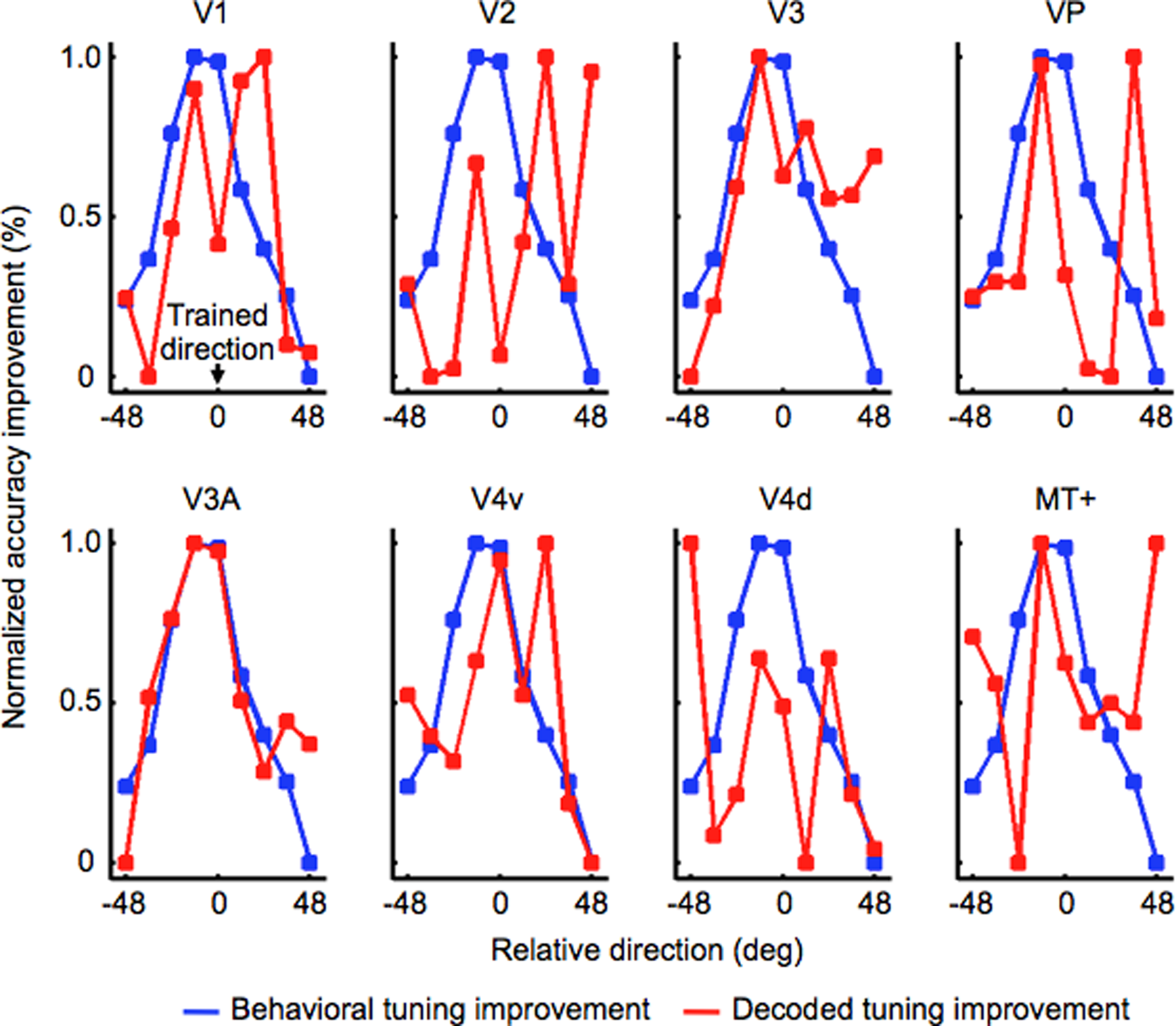

Supplement: Figure S5 — Comparison between the behavioral (blue) and decoded (red) tuning improvement functions for each ROI. Each improvement function was scaled from 0 to 1 for visualization purpose. Only for V3A, a significant correlation was found (r = 0.86, P<0.05, false discovery rate, corrected by the number of the ROIs), but not for the other ROIs (P>0.11, no multiple correction). (TIFF) [file pone.0044003.s005.tif]

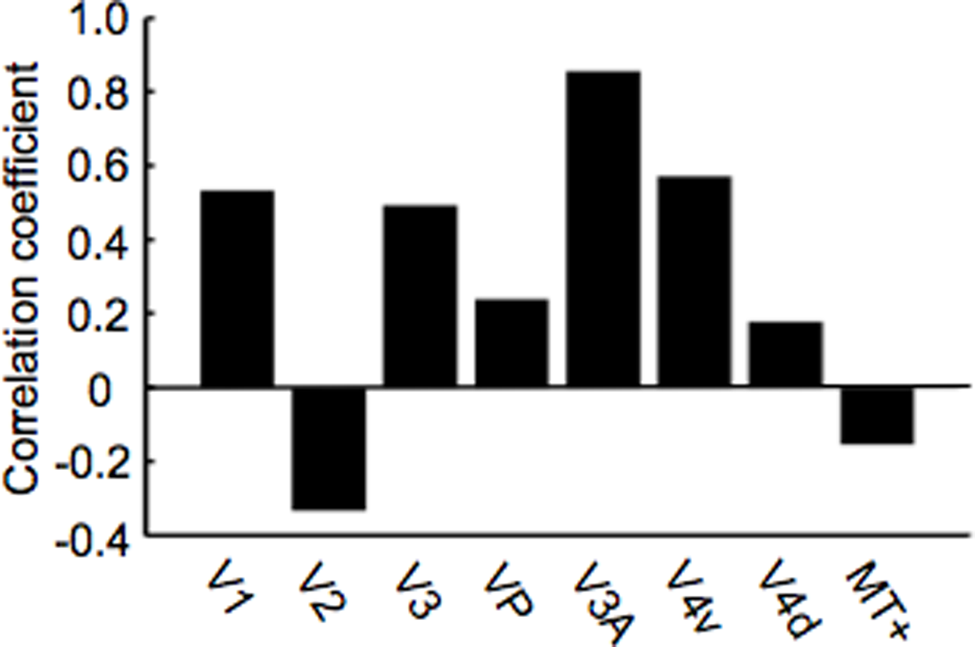

Supplement: Figure S6 — The correlation coefficient between the behavioral and decoded tuning improvement functions for each ROI. (TIFF) [file pone.0044003.s006.tif]
